# Born Too Early and Too Small: Higher Order Cognitive Function and Brain at Risk at Ages 8–16

**DOI:** 10.3389/fpsyg.2019.01942

**Published:** 2019-09-06

**Authors:** Marta Córcoles-Parada, Rocio Giménez-Mateo, Victor Serrano-del-Pueblo, Leidy López, Elena Pérez-Hernández, Francisco Mansilla, Andres Martínez, Ignacio Onsurbe, Paloma San Roman, Mar Ubero-Martinez, Jonathan D. Clayden, Chris A. Clark, Mónica Muñoz-López

**Affiliations:** ^1^Human Neuroanatomy Laboratory, School of Medicine and Regional Centre for Biomedical Research, University of Castilla-La Mancha, Albacete, Spain; ^2^Department of Psychology, University of Area Andina, Bogotá, Colombia; ^3^Developmental and Educational Psychology, Autonomous University, Madrid, Spain; ^4^Radiology Service, Sta. Cristina Clinic and University Hospital of Albacete, Albacete, Spain; ^5^Neonatology Service, University Hospital of Albacete, Albacete, Spain; ^6^Paediatric Neurology Service, University Hospital of Albacete, Albacete, Spain; ^7^Child Psychiatry Service, University Hospital of Albacete, Albacete, Spain; ^8^Department of Anatomy, Catholic University of Murcia, Murcia, Spain; ^9^Developmental Imaging and Biophysics Section, Institute of Child Health, University College London, London, United Kingdom; ^10^Centre for Clinical Brain Sciences, University of Edinburgh, Edinburgh, United Kingdom

**Keywords:** preterm, perinatal clinical variables, cognition, MRI, DTI, cognitive function, fornix, memory

## Abstract

Prematurity presents a risk for higher order cognitive functions. Some of these deficits manifest later in development, when these functions are expected to mature. However, the causes and consequences of prematurity are still unclear. We conducted a longitudinal study to first identify clinical predictors of ultrasound brain abnormalities in 196 children born very preterm (VP; gestational age ≤32 weeks) and with very low birth weight (VLBW; birth weight ≤1500 g). At ages 8–16, the subset of VP-VLBW children without neurological findings (124) were invited for a neuropsychological assessment and an MRI scan (41 accepted). Of these, 29 met a rigorous criterion for MRI quality and an age, and gender-matched control group (*n* = 14) was included in this study. The key findings in the VP-VLBW neonates were: (a) 37% of the VP-VLBW neonates had ultrasound brain abnormalities; (b) gestational age and birth weight collectively with hospital course (i.e., days in hospital, neonatal intensive care, mechanical ventilation and with oxygen therapy, surgeries, and retinopathy of prematurity) predicted ultrasound brain abnormalities. At ages 8–16, VP-VLBW children showed: a) lower intelligent quotient (IQ) and executive function; b) decreased gray and white matter (WM) integrity; (c) IQ correlated negatively with cortical thickness in higher order processing cortical areas. In conclusion, our data indicate that facets of executive function and IQ are the most affected in VP-VLBW children likely due to altered higher order cortical areas and underlying WM.

## Introduction

Despite increased survival and decreased morbidity of children born very premature (VP) with very low birth weight (VLBW), the long-term outcome of these children is variable. Recognition of the risks and consequences is a concern through the world (World Health Organization, 2018). This concern is extensive from children born VP-VLBW who develop with brain and neurological deficits to those, the great majority nowadays, that instead grow with higher order cognitive difficulties. The high incidence of these subtler cognitive difficulties often apparent later in development, i.e., at school age and/or teenager hood and has been indirectly evidenced in large cohort studies – EPIPAGE (Larroque et al., 2008) and EPICURE (Moore et al., 2012) – revealing that about one third of children born preterm seek specialist assistance later in life, especially in psychiatry and educational services. Common reported concerns are poorer intelligence quotient (IQ), working memory, attention, and executive function (Nosarti et al., 2008; Geldof et al., 2013; Kalpakidou et al., 2014; Eryigit Madzwamuse et al., 2015; Murray et al., 2016; Franz et al., 2018; Loe et al., 2018; Aanes et al., 2019). Such deficits, even if subtle, may hinder academic achievement as well as integration in society. It remains unclear, however, what specific aspects of these functions are associated with changes in specific brain areas or networks.

The first step has been to find out what modifiable risk factors impact cognitive development of children born VP-VLBW; with the consequent optimization of early interventions (Pierrat et al., 2017). The second key step is to characterize the cognitive and brain profile of children born VP-VLBW. One way of doing this is by measuring cognitive function in association with structural and functional magnetic resonance imaging (MRI). Structural brain morphometric data of VP-VLBW children indicate that, at school age, preterm children show changes in cortical gray and white matter (WM) volumes, as well as in surface area in sensorimotor cortex and higher order processing areas of the cerebral cortex such as the prefrontal, posterior superior temporal and parietal and occipital cortices (Skranes et al., 2013; Akazawa et al., 2015; Sølsnes et al., 2015; Zhang et al., 2015). Not only the cerebral cortex seems to be affected in prematurity but also the thalamus, cerebellar WM (Martinussen et al., 2009; Brossard-Racine et al., 2017), the *globus pallidus*, and the *corpus callosum* (cc) (Malavolti et al., 2017; Lean et al., 2019). There is also an enlargement of the lateral ventricles in adolescence and early adulthood (Bjuland et al., 2014). This data support the notion that, in addition to sensoriomotor difficulties, higher order cognitive function is at risk in very preterm children. Brain connectivity data from diffusion tensor imaging (DTI) (Anjari et al., 2007; Rose et al., 2008; Duerden et al., 2019), tractography (Murray et al., 2016), and more recently, connectome analysis (Thompson et al., 2016) points to altered WM connectivity in preterm children compared with infants born at term. There are also recent studies trying to assess DTI parameters in fetuses, which can be useful in the future to study abnormalities *in utero* as well as the impact the exposure to the ex utero environment on brain development may have (Mitter et al., 2015; Lockwood Estrin et al., 2019). However, despite of the flourishing studies on human brain imaging, clinical data, or animal models, the problem of risks, mechanisms, and neurocognitive consequences of being born too early still escape our hands.

To start addressing some of the causes and consequences of neurocognitive impairment in preterm children, we designed a two phases study. Phase I aimed to identify causes of brain damage in VP-VLBW neonates by looking at the association of perinatal risk factors and ultrasound brain abnormalities detected in new-born VP-VLBW babies (i.e., gestational age, GA ≤ 32 weeks, and birth weight, BW ≤ 1500 g). Our hypothesis is that some specific perinatal factors explain, at least in part, the variability in neonatal brain abnormalities in VP-VLBW neonates. Phase II was designed to determine neurocognitive outcome of high order cognitive functions in a subgroup of neurologically healthy children from the VP-VLBW cohort at school age (8–16 years old). With this aim, we used restrictive selection criteria to include only those children that were VP-VLBW and showed no brain abnormalities in MRI and no neurological signs. Our hypothesis is that higher order cognitive functions such as executive function and memory are affected in association with altered higher order processing areas of the cerebral cortex and underlying WM in the VP-VLBW group.

## Materials and Methods

### Participants

#### Neonates

For Phase I, preterm babies from total live births from 01/01/95 to 31/12/04 born/referred at born to the Albacete University Hospital were included. Inclusion criteria were GA ≤ 32 weeks and BW ≤ 1500g. Chromosomal syndromes, often associated with cardiac, respiratory, and/or brain abnormalities, could be confounding factors in this study, and therefore, children diagnosed with such syndromes were excluded.

#### School Age (8–16 Years Old)

In Phase II, children from the previous group who had no neurological or MRI findings were invited to take part at 8–16 years of age. An age and gender matched control group was recruited in schools in the same demographic area.

### Ethics Statement

This study was conducted according with the World Medical Association ethical principles for research (Rickham, 1964) and with the local ethical University of Castilla-La Mancha Medical School and the Albacete University Hospital clinical research committee (Acta 02/12). Written and signed informed consent was obtained for all the participants and their parents.

### Perinatal Variables and Brain Ultrasonography at Birth

To identify risk factors for abnormal brain ultrasonography (Phase I of the study), we examined medical charts of all the VP-VLBW children. Table 1 defines the neonatal variables included in this study. Brain ultrasonography was performed with a General Electric LOGIA 400 CL ProSeries system at birth and/or before the end of the first week of life. Brain ultrasound abnormalities were diagnosed by a pediatrician (AM) and a neuroradiologist (FM).

**TABLE 1:** Neonatal clinical variables included in this study.

**Variables**	**Definition**
**Mother/pregnancy**	
Toxic habits	Smoking
Reproductive/Gynecological	Fertilization treatments, previous preterm births
Medical condition	Diabetes mellitus, hypertension

**Intrapartum**	
Amniorrhexis	Prelabor rupture of membranes.
Pre-eclampsia	Hypertension, proteinuria, and edema after the 20th week of gestation
Mode of delivery	Normal vaginal, cesarean, emergency cesarean, elective cesarean
Fetal presentation	Unknown, cephalic, podalic, bottom, transverse

**Neonatal**	
Gestational age (weeks)	Positive ultrasonography, last menstrual period, clinical assessments
Apgar scores at 1′ and 5′	Scores from 1 to 2 of Appearance, Pulse, Grimace, Activity, Respiration

**Respiratory**	
Respiratory distress	Bronchopulmonary dysplasia, oxygen dependency, apnea, hyaline membrane disease
Bradycardia	Heart rate <100 l pm
Pulmonary hemorrhage	Positive bronquial aspiration for blood and respiratory deterioration
Pulmonary hypertension	Superior right limb and left inferior limb gradient in oxygen saturation >5% + hypoxemic trend
Pneumothorax	Thorax x-ray confirmation of air in pleural space
Asphyxia	Metabolic acidosis in umbilical cord pH + neonatal encephalopathy + background of alterations in cardiotocographic fetal exploration

**Cardiac hemodynamic**	
Systolic murmur	Positive auscultation for heart murmur
Patent *ductus arteriosus*	Sistolc murmur + tensional gradient + bounding pulses and respiratory deterioration. Arterial flow between aorta and pulmonary artery shown via bidimensional echocardiography
Hypertension	Systolic pressure higher than percentile 90 Hg for gestational age
Hypotension	Mean arterial blood pressure lower than the gestational age during the first 3 days
Jaundice of prematurity	Increased bilirubinemia over birth weight expressed in g/100
Anemia	Level of hemoglobin in blood count <12 g/dl
Thrombocytopenia	Level of platelets <100.000/mm3
Coagulopathy	Altered of activated partial thromboplastine time or prothrombine time

**Ultrasound brain abnormalities**	
Periventricular leukomalacia	Unilateral/bilateral hypo-echogenicity areas in brain parenquima adjacent to lateral boundaries of the ventricular system.
IV hemorrhage	Unilateral/bilateral abnormally extensive hypo-echogenicity areas in the ventricular system
Hydrocephalus	Symmetric bilateral increased size of the ventricular system
Microcephaly	<Percentile 10

**Neurological**	
Cerebral palsy	Chronic disability of central nervous system origin characterized by aberrant control of movement and posture, appearing early in life
Severe mental retardation	Severely retarded individuals have IQ scores of <40
Seizures/Epilepsy	Presence of convulsive movements with or without electroencephalography correlation
Tetraplegia, diplegia	Paralysis affecting the four limbs, paralysis affecting symmetrical parts of the body

**Inflammatory**	
Sepsis	Clinically, symptoms and alterations from laboratory results compatibles with infection with negative cultures. Confirmed, symptoms of infection + blood culture or urine culture and/or positive cerebrospinal fluid
Necrotizing enterocolitis	Digestive tolerance alteration necrotizing enterocolitis type II or higher
Otitis	Otic pain + positive symptom with compatible eardrum exploration
Conjunctivitis	Presence of conjunctival pus drainage

**Ophthalmic**	
Retinopathy of prematurity	Defined according to the criteria established by the Committee for the Classification of Retinopathy of Prematurity (The International Classification of Retinopathy of Prematurity| Neonatology | JAMA Ophthalmology | The JAMA Network)

**Medical treatments**	
Red blood cell transfusion	Hemoglobin level below 10 g/dL or hematocrit falls below 25%
Oxygen therapy	Oxygen is applied up to get saturations of 90–95% in pulse oxymetry
Mechanical ventilation	Mechanically assist or replace spontaneous breathing
Surgery	Any surgery during hospitalization

### Neuropsychological Assessment and MRI Scan

In Phase II, the 8–16 years old VP-VLBW and the control group were assessed by the pediatric neurologist and had an MRI scan before being assessed with a neuropsychological battery of tests.

The battery of standardized neuropsychological tests assessed: intelligence (Wechsler Intelligence Scale for Children, WISC-IV) (Wechsler, 2003), general memory (Test of Memory and Learning, TOMAL) (Reynolds and Bigler, 1994), spatial and everyday memory (Rivermead Behavioral Memory Test, RBMT) (Wilson et al., 1985), attentional interference (STROOP) (Golden, 1978), and verbal fluency (Children Executive Functions, ENFEN) (Portellano et al., 2009). Selective attention, impulsivity, and vigilance were tested with a computerized test (Continuous Performance Test, CPT-II) (Conners, 2000). Parents, participants and teachers filled in the questionnaire Behavioral Assessment System for Children (BASC) (Reynolds and Kamphaus, 1992). Parents also filled a socioeconomic questionnaire specifically created for this study to register annual parental income and academic degree.

### Statistics

Statistical analysis (SPSS, v.20.0) was conducted for both, neonates and the 8–16 years old group.

In Phase I, neonatal clinical data was first reduced by means of a principal component analysis (PCA) stepwise backward. Second, to identify possible associations between neonatal variables and presence of ultrasound brain abnormalities, a multivariate logistic regression analysis was calculated with maximum likelihood model estimation (*p* ≤ 0.05 and 95% confidence interval). Presence/absence of ultrasound brain abnormality was considered as dependent variable and the factors obtained in the PCA analysis were included as independent variables. *p* ≤ 0.05 was considered significant for all the analyses.

In Phase II, neuropsychological data of the 8–16 years old participants was analyzed with multivariate analysis of variance (ANOVA) tests or Mann-Witney *U* tests. Kolmogorov-Smirnov and Shapiro-Wilk normality tests were used. Significant differences were taken if *p* ≤ 0.05.

### Brain Imaging

#### MRI Acquisition at 8–16 Years of Age

Phase II participants’ (ages 8–16) were scanned in an Optima MR 450 W General Electric 1.5T scanner with: (1) anatomical SPGR 3D, TR: 8.5 ms, TE: 3.2 ms, TI: 400 ms, thickness: 1 mm, gap: 0, isotropic matrix: 256 × 256 × 256, nex: 1, acquisition time = 7.28 min; (2) Diffusion Tensor Imaging: 20 directions, TR: 9425 ms, TE: 103 ms, angle: 90°, thickness: 3, gap: 0, nex: 2, voxel size: 3 × 3 × 3, acquisition time = 6.45 min. A clinical neuroradiologist (FM) reported no significant findings. MRI data pre-processing started by converting the DICOM images into NIfTI format using dcm2nii tool from MRIcron (Rorden and Brett, 2000).

#### Brain Gray Matter Integrity

Cortical reconstruction and volumetric segmentation of gray matter (GM) was performed with Freesurfer (version 6.0.0^1^) (Dale et al., 1999; Fischl et al., 1999a,b, 2001, 2002, 2004a,b; Ségonne et al., 2004). Accuracy of automated segmentation was reviewed and control points were used according with Freesurfer guidelines.

Cortical surface was segmented according with the Desikan-Killiany atlas (Desikan et al., 2006). Surface area was the sum of the area of the vertices in each parcellation; cortical thickness was calculated as the closest distance from the WM/GM boundary to the pial surface at each vertex on the tessellated surface (Fischl and Dale, 2000). Volume was the product of the surface area by cortical thickness for each region. In the automatic subcortical segmentation, each voxel in the normalized brain volume was assigned to one of about 40 labels, determined by location and intensity.

Cortical thickness analysis was run by using the single-binary application included in Freesurfer QDEC 1.5. Multivariate analysis of variance (ANCOVA, SPSS) was used for surface area, cortical and subcortical GM volume with intracranial volume (ICV), gender, and age at scan as covariates.

Spatial correlations were run between cortical thickness and neuropsychological values below control levels in the VP-VLBW group. General linear model in QDEC 1.5 was used, controlling for ICV, gender, and age. SPSS v.20.0 was used for the correlations between cortical surface area and GM volume with the cortical and subcortical parcellations and neuropsychological scores. Bonferroni multiple was used for correction. Significant differences were taken if *p* ≤ 0.05.

#### Brain White Matter Integrity

##### Tract-based spatial statistics (TBSS)

The pipeline for DTI data processing was conducted with the FMRIB’s Diffusion Toolbox (FDT, FSL, FMRIB Software Library, Oxford, United Kingdom). The DTI images were registered to the non-diffusion-weighted (b0) image to minimize artifacts due to eddy currents distortions, and a mask was created to exclude non-brain voxels. The skull was then removed from the image using the FSL BET tool. Fractional anisotropy (FA), mean diffusivity (MD), and λ1, λ2, and λ3 values were calculated by using the FSL DTI-FIT to fit all the tensors. λ1 is considered axial diffusivity (AD). λ2 and λ3 values were averaged to obtain radial diffusivity (RD) maps.

Participants’ FA maps were aligned to a 1 × 1 × 1 mm standard space by a non-linear registration and the FMRIB58 image as a target. The aligned images were used to create a mean FA map and a mean FA skeleton, with the centers of all the tracts common to the group. This FA skeleton was thresholded at FA >0.2 to include the major WM pathways and exclude most of the GM. Then, each subject’s aligned FA data were projected onto this skeleton and the resulting data were fed into it voxelwise across subjects. Multiple error correction was controlled with the randomize tool, and FSL’s TFCE was used to carry out non-parametric permutation-based statistical comparisons of VP-VLBW vs. control FA, MD, AD, and RD maps (5000 permutations) with ICV, gender, and age at scan as covariates. Significance if *p* ≤ 0.05.

Whole brain FA and RD as well as the anatomical tracts were investigated in terms of correlation analysis with neuropsychological data.

## Results

### Neonates

In Phase I of the study, of the 36,001 live births at the Albacete University Hospital, approximately 8% were preterm and 1% were VP-VLBW. Of the 270 (0.7%) potentially eligible children, 207 (77%) survived. Causes of demise were: cardiac arrest (27), asphyxia (23), septic shock (10), and multiple malformation syndrome (3). Of the 207 survivors, 4 had chromosomal malformations and 7 had incomplete charts and were excluded. The final sample that forms the VP-VLBW cohort was of 196 babies. Neonatal data of all the VP-VLBW children included is summarized in Table 2.

**TABLE 2:** Neonatal data of babies born very preterm/very low birth weight with presence/absence of ultrasound brain abnormalities. Summary of results from the principal component (PCA) and the multivariate logistic regression analyses.

		**Brain abnormality (n = *72)***	**No brain abnormality (*n* = *124)***		

**Neonatal data**	***n***	**Median *(IQR)***	**Median *(IQR)***	**U Mann Whitney**	**Cohen’s d value**
Gestational age (weeks)	196	29 (28–31)	30 (28–31)	3733.50^∗^	0.33
Birth weight (g)	196	1126 (950–1300)	1225 (1075–1368)	3472.00^∗∗^	0.38
Body length (cm)	196	37 (35–39.4)	38 (36.6–40)	3822.50	0.27
Head circumference (cm)	196	27 (25–28)	27 (26–28)	3714.00^∗^	0.33
Apgar 1′	196	6 (4.2–8)	8 (6–8)	3417.00^∗∗^	0.47
Apgar 5′	196	8 (7–9)	9 (8–9)	3206.50^∗∗∗^	0.40
Mechanical ventilation	196	6.50 (1–14.5)	1 (0–4)	2588.00^∗∗∗^	−0.73
Oxygen therapy	196	20 (3–53)	8 (2–30)	3528.50^∗^	−0.54
Hospitalization	196	65.50 (51.25–88)	50 (40.25–59)	2519.50^∗∗∗^	−0.75
Neon. intensive care unit	196	31.50 (18–53.25)	19.50 (10.25–29.75)	2724.50^∗∗∗^	−0.74

		***N (%)***	***N (%)***	**χ^2^_(1)_**	***OR-CI***

Hypotension	195	27 (19)	13 (16)	5.89^∗∗^	0.41–0.19–0.85^∗^
Asphyxia	196	11 (8)	1 (1)	11.04^∗∗∗^	0.07–0.01–0.53^∗∗^
Resp. distress	196	82 (59)	65 (81)	6.16^∗∗^	0.42–0.21–0.84^∗^
Pneumothorax	195	6 (4)	0	7.13^∗∗^	0.94–0.90–0.99^∗∗^
Necrotizing enterocolitis	196	26 (19)	10 (12)	9.55^∗∗^	0.3–0.14–0.66^∗∗^
Retinop. of prematurity	196	44 (32)	23 (29)	9.42^∗∗^	0.38–0.21–0.71^∗∗^

**Principal component analysis**		**PCA weight**	**Explained variance (%)**	***OR (95% CI)***	***p***

*PC 1 – Post-birth hospitalization*			30.586	3.08 (1.89–4.99)	0.000^∗∗^
Hospitalization (days)		0.866			
Neonatal intens. care unit (days)		0.856			
Mechanical ventilation (days)		0.825			
Oxygen therapy (days)		0.679			
Surgeries		0.602			
Retinopathy of prematurity		0.574			
*PC 2 - Apgars*			11.290	0.61 (0.43–0.87)	0.006^∗∗^
Apgar 1′		0.905			
Apgar 5′		0.864			
*PC 3 – Respiratory distress and ventilation*			8.597	1.34 (0.95–1.89)	0.096
Respiratory distress		0.856			
Mechanical ventilation (freq.)		0.651			
*PC 4 – Gestational and severe respiratory problems*			6.815	2.37 (1.23–4.55)	0.010^∗^
Gestational age (weeks)					
Birth weight (g)					
Asphyxia		0.749			
Pneumothorax		0.689			
*PC 5 – NEC and hemodynamic*			6.399	1.58 (1.09–2.27)	0.015^∗^
Necrotizing enterocolitis		0.799			
Hypotension		0.566			
*PC 6 – Fertilization treatments*			5.992	0.69 (0.45–1.05)	0.083
*In vitro* fertilization		0.916			

*^∗^*p* ≤ 0.05, ^∗∗^*p* ≤ 0.01, ^∗∗∗^*p* ≤ 0.001.*

#### Neonatal Ultrasound Brain and Neurological Findings

Within the VPLBW new-born babies, 72 (37% of 196) had one type of ultrasound brain abnormality. Intraventricular hemorrhage was the most common (20 cases), followed by choroid plexus pathology (13), microcephalia (6), periventricular leukomalacia (5), and hydrocephalus (2). The remaining children (26) had more than one finding. Convulsions without any ultrasonography abnormality were seen in 6 cases.

Of the 72 babies with brain findings, 18 had severe neurological consequences defined by the neurological pediatrician and the neonatologist: 6 were diagnosed with one problem only (cerebral palsy, severe mental retardation, epilepsy, blindness, deafness, encephalopathy) whereas 12 presented multiple diagnoses.

#### Data Reduction and Multivariate Logistic Regression

The PCA reduced the 17 neonatal variables that discriminated those children with brain ultrasound abnormalities into 6 components. These PCAs explained up to 70% of the variance. Table 2 illustrates the PCAs extracted (PCA, row headings) together with the results from the multivariate logistic regression (column headings). The first (Table 2, *PCA 1 – Post-birth hospitalization*) and second factors (Apgars) carried the main weight, 30 and 11%, respectively and together explain most of the variability. Gestational age, birth weight and severe respiratory factors explain less variability, but were effective predictors of ultrasound brain abnormalities. The logistic regression showed that post-birth hospitalization course [Exp(B) = 3.08; *p* < 0.001], apgars (Exp(B) = 0.61; *p* < 0.001), gestational and severe respiratory problems [i.e., asphyxia and pneumothorax; Exp(B) = 2.37; *p* < 0.01], and NEC and hemodynamic [Exp(B) = 1.58; *p* < 0.05] were significant predictors of ultrasound brain abnormalities [constant Exp (B) = 0.584; *p* < 0.01]. On the other hand, respiratory distress, ventilation and *in vitro* fertilization, although more frequent in the group with brain injury, were less determinant of brain injury.

### School Aged Children (8–16 Years Old)

For Phase II, 124 children were eligible and 41 (33%) agreed to participate. Of them, 2 had no MRI scan, 2 no neuropsychological assessment, and 8 had poor quality MRI due to motion artifacts, leaving a final sample of 29 VP-VLBW children (16 males, 55%, mean age at scan = 11.38, SD = 2.82). Within the initial control group (*n* = 20), 6 had poor quality MRI, so 14 participants formed the final control group (9 males, 64%, mean age at scan = 10.90, SD = 2.67). Both VP-VLBW (*n* = 29) and control groups (*n* = 14) had similar ages at scan (*t*_41_ = −0.50, *p* = 0.62), similar gender proportion (*X*_1_^2^ = 0.32 *p* = 0.74), and similar parental annual family income (*t*_41_ = 1.62, *p* = 0.13).

#### Neuropsychological Results at Ages 8–16

At age 8–16, VP-VLBW children’s mean full scale IQ (WISC) was 11 points below control levels, with perceptual reasoning index especially affected (see Table 3). Mean scores in phonological fluency (ENFEN; executive function), were lower in the target group relative to control levels. Teachers reported learning difficulties (BASC-T) and poorer academic results in the VP-VLBW group. Nevertheless, the VP-VLBW had intact selective attention, impulsivity, vigilance, and resistance to (STROOP) interference, as well as spatial, everyday memory, and the different forms of recognition, learning and delayed memory assessed by the general memory and learning test TOMAL. However, the TOMAL test revealed poor performance in two measures of working-memory – immediate memory for stories and inverse letter sequencing.

**TABLE 3:** Neuropsychological and gray matter volume (mm^3^) differences in control and preterm groups.

**Test**	**Control *(n* = *14)***	**Preterm *(n* = *29)***	***F_1__,__41_(p)***
	**Mean *(SEM)***	**Mean *(SEM)***	
*Intelligence (WISC IV)*			
Full IQ	112.57 (3.83)	101.69 (3.06)	4.46 (0.04)^∗^
Perceptual reasoning	109.07 (4.06)	96.97 (3.18)	5.07 (0.03)^∗^
Block design^1^	10.93 (0.67)	8.66 (0.63)	4.96 (0.03)^∗^
Information^1^	13.00 (0.59)	10.55 (0.58)	6.80 (0.01)^∗∗^
Arithmetic^1^	12.86 (0.67)	9.59 (0.70)	8.58 (0.01)^∗∗^
*General memory (TOMAL)*			
Immediate memory for stories^1^	14.29 (0.69)	11.93 (0.55)	6.41 (0.02)^∗^
Letters backward^1^	12.00 (1.14)	9.41 (0.48)	6.09 (0.02)^∗^
*Executive function (ENFEN)*			
Phonological fluency^1,2^	0.05 (0.25)	−1.03 (0.32)	4.09 (0.01)^∗∗^

**Brain region**			

*Left*			
Thalamus	8247.97 (907.33)	7362.94 (563.73)	12.03 (0.001)
Lateral ventricle	5684.77 (2730.78)	10117.43 (5871.07)	10.77 (0.002)
Ventral diencephalon^*T*^	4038.12 (506.15)	3559.78 (282.80)	14.14 (0.001)
*Right*			
Thalamus	7987.00 (884.50)	7043.21 (559.03)	15.01 (≤ 0.001)
Fusiform gyrus cortex	11071.00 (1393.64)	9936.41 (1011.79)	17.21 (≤ 0.001)
Entorhinal cortex area (mm^2^)	374.28 (75.14)	458.28 (67.49)	15.09 (≤ 0.001)
			

*^1^Subtests, ^2^Standard *z* scores, ^*T*^Ventral diencephalon in FreeSurfer includes: hypothalamus, basal forebrain, sublenticular extended amygdala and ventral tegmentum (although the last one is part of the midbrain). ^∗^*p* ≤ 0.05, ^∗∗^*p* ≤ 0.01.*

In sum, working and verbal immediate memory appear to be below control levels as well as general cognitive abilities measured by the intelligence test. The impairments, even if mild, may preclude learning difficulties as reported by the teachers (BASC-T, VP-VLBW mean = 51.54 ± 8.15; control = 45.25 ± 10.45; *p* = 0.07). These children’s attention and spatial and everyday memory were at control levels.

#### Brain Gray Matter Integrity

A 5.7%, decreased total GM and 10% subcortical GM volumes (*F* = 8.68, *p* < 0.01; *F* = 6.91, *p* < 0.05, respectively) were observed in the VP-VLBW group relative to control levels. After Bonferroni correction for multiple comparisons, significant decreased volumes were found bilaterally in the left and right thalamus, and left ventral diencephalon including hypothalamus, basal forebrain, sublenticular extended amygdala, and ventral tegmentum (although the last three are not part of the diencephalon, Freesurfer software includes them together as part of it) in the VP-VLBW group. This group had significant decreased cortical volume in right fusiform gyrus and enlarged left lateral ventricle relative to control levels, but no correlations with neuropsychological variables were observed. Surface cortical area in children VP-VLBW was increased in right entorhinal cortex. Values for volumes and areas are reported in Table 3.

**FIGURE 1:**
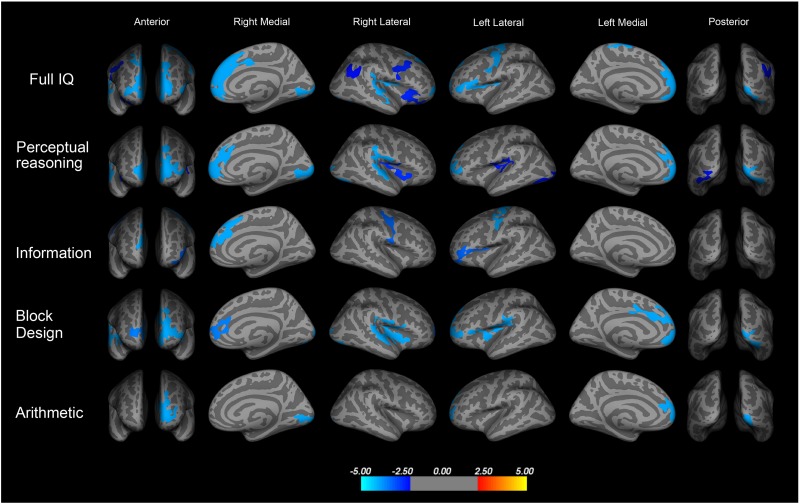
Spatial negative correlation of cortical thickness and intelligence quotient (IQ) in children born very preterm and with VLBW at ages 8–16 (*p* ≤ 0.05, corrected for ICV, age, and gender). Thickness of high order processing cortical areas (in blue) was associated with full scale IQ, specifically with perceptual reasoning index, and the subscales of information, block design, and arithmetic. Perceptual reasoning: frontal pole, mPFC (areas 24, 32, and 9), insula, posterior superior temporal gyrus, right inferior parietal cortex and extra striate visual cortex; Information: left inferior frontal and superior frontal gyrus, sensory and motor areas; Block design: left frontal pole and anterior cingulate cortex (area 24), superior medial frontal gyrus (areas 32 and 8/9), left inferior parietal cortex, and left extrastriate visual cortex, right posterior superior temporal gyrus, and insula; Arithmetic: left frontal pole and extrastriate visual cortex (*unpublished data*).

No differences in global cortical thickness were found, but cortical thickness in several brain areas was negatively correlated with full scale IQ, so the lower the IQ the thicker the cortex (see spatial correlation in Figure 1). Furthermore, cortical thickness was also negatively correlated with the perceptual reasoning index and the subscales of information, block design, and arithmetic in the VP-VLBW group.

As illustrated in Figure 1, full scale IQ correlated negatively with thickening of frontal pole, medial prefrontal cortex (mPFC, approximately in areas 32 and 8/9), and anterior cingulate cortex (approximately area 24), left (posterior) inferior frontal gyrus, sensory and motor areas, dorsal and posterior insular cortex, posterior superior temporal gyrus, and extrastriate visual cortex. However, the neonatal clinical data had not predictive value in terms of prediction for the neuropsychological nor brain imaging parameter.

#### Brain White Matter Integrity

**FIGURE 2:**
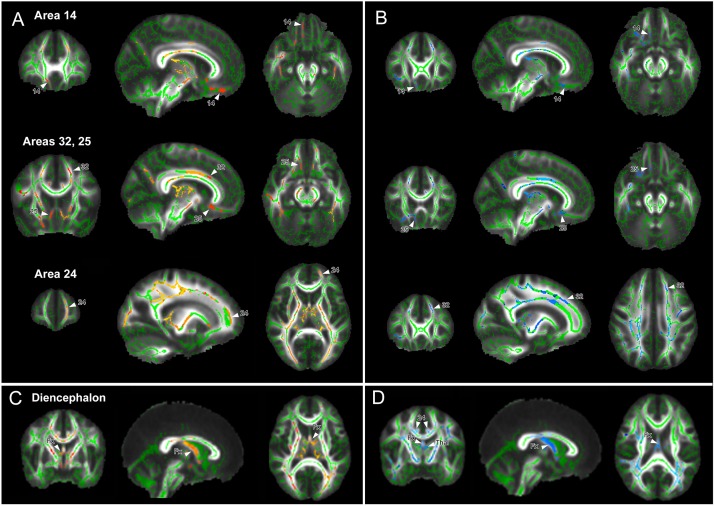
White matter regions with significant changes in fractional anisotropy (FA) and radial diffusivity (RD) after correction for intracranial volume, age, and gender (*p* ≤ 0.05). **(A)** Cortical regions with decreased FA (red-yellow) and **(B)** with increased RD (blue) in the very preterm/very low birth weight group. Decreased FA/increased RD was found in medial frontal areas, temporal, and extrastriate visual and parietal white matter. **(C)** Significant decreased FA (red-yellow) and **(D)** increased RD (blue) in diencephalon, including the fornix (Fx) and thalamic white matter (Thal) in children in the very preterm/very low birth weight group.

After correction for multiple comparisons, significant differences in WM voxels between groups (*p* < 0.05) are shown in Figure 2. According to the segmentation from previous studies (Córcoles-Parada et al., 2017), results showed significant decreased FA values in preterm born children in the WM of mPFC areas 14, 24, 32, and 25 (Figure 2A in red-yellow), and diencephalic WM (Figure 2C). In addition, decreased FA was observed in the medial parietal WM, pyramidal tract, and optic radiation. None of the WM areas showed significant increase in FA in the preterm group relative to the term-born group.

Voxels with significant increased RD values overlapped with regions of decreased FA values (Figures 2B,D, in blue) except for the medial parietal region, where this overlap is less evident.

## Discussion

Here we examined a cohort of children who were both born with less than 32 gestational weeks and less than 1500g as babies and at a later stage in development; i.e., 8–16 years of age. This cohort study had a percentage of preterm babies (8%) like that reported by other countries, with circa 1% born with same gestation and birth weight. Data presented here confirmed that one third of the babies had ultrasound brain abnormalities and, of those, still a quarter had severe neurological problems. Those children with ultrasound brain abnormalities had lower GA, birth weight, cranial perimeter, and Apgars; together with hospitalization course (i.e., hypotension, necrotizing enterocolitis, retinopathy of prematurity), and severe respiratory complications such as asphyxia and pneumothorax. This confirms previous data on the frequent postnatal comorbidities of the preterm infant (Wheater and Rennie, 2000; Schmidt et al., 2003; Becher et al., 2004; Hintz et al., 2005; Schulzke et al., 2007). As several variables seem to interact and, it seems difficult to predict neonatal brain injury from a specific clinical risk factor (Becher et al., 2004; Limperopoulos et al., 2007; Thompson et al., 2019).

Subtle neurodevelopmental deficits are difficult, if not impossible, to detect at such an early stage - one basis for looking at much older children in the second part of the study.

### Neuropsychological Consequences of Prematurity at 8–16 Years Old

Children born with very low birth weight (VP-VLBW), but who could be considered healthy as they had neither brain injuries detectable by ultrasound at birth or MRI later in life, however: a) not only VP-VLBW children had IQs below control levels, but, importantly, cortical thickness in higher order processing areas of the cerebral cortex was correlated negatively with IQ; and b) they had deficits in immediate memory (immediate memory for stories and letters backward) and executive function (verbal fluency), but spared basic attentional functions and memory (general, spatial and episodic). These impairments, even if mild, seem to hinder school results and learning levels as reported by the teachers.

#### Intelligence Quotient

The lower IQ in the VP-VLBW group in this study is in agreement with previous reports (Isaacs et al., 2003), even when applying different methods (Tideman, 2000; Weisglas-Kuperus et al., 2009). Within the WISC-IV subtests, our study detected deficits in the preterm children in arithmetic, information, and block design, which is in agreement with previous studies (Hack et al., 2002; Tulsky, 2003; Kulseng et al., 2006; Taylor et al., 2009; Løhaugen et al., 2010). In particular, the arithmetic subtest has been described as a multifaceted test that requires mathematical and working memory skills among others and in our study were the ones that showed worst outcome (Tulsky, 2003). Mathematical problems have been previously found to be especially common in preterm children, even when controlling for IQ and neurodevelopmental disorders (Taylor et al., 2009). Statistical differences on the block design subtest, which seems to be one of the most sensitive subtests to clinical conditions, are also in accordance with previous findings (Hack et al., 2002; Tulsky, 2003; Kulseng et al., 2006). No further significant differences were observed when participants were re-grouped by age (≤11 vs. >12 years), indicating that age was not a confounding variable in this study.

#### Attention

Although attentional deficits are often diagnosed in preterm cohorts (Bhutta et al., 2002; Rommel et al., 2019), there are variable findings when profiling the impaired subdomains in preterm cohorts with some reporting deficits in only some of the attentional networks (Bayless and Stevenson, 2007; Fazzi et al., 2009; Mulder et al., 2011; Cserjesi et al., 2012). In our study, some of the basic components of attention were preserved in the VP-VLBW group. However, they had poorer immediate memory and phonological fluency relative to control levels, suggesting that higher order executive functions, likely dependent on prefrontal connectivity with cortical (temporal, parietal) and subcortical structures (diencephalon, striatum), had been affected.

#### General and Episodic Memory

Other deficit usually reported in preterm children are episodic-like memory problems associated with hippocampal damage (Isaacs et al., 2001; Giménez et al., 2004; Rose et al., 2011; Aanes et al., 2019; Strahle et al., 2019). There is, however, recent controversy (Brunnemann et al., 2013; Omizzolo et al., 2013; Thompson et al., 2014). This contradictory data is likely to be related with the magnitude of the decrease in hippocampal volumes in preterm babies – from 10 to 16%, with only occasional cases with 20% volume reduction or over. In our study, general memory (TOMAL), spatial, and everyday memory (Rivermead Behavioral Memory Test) were spared. In addition, we observed no reduction in hippocampal volume (data not shown) and no correlations were found between hippocampal volume and neuropsychological variables. However, our cohort of children VPT-VLBW did have reduced immediate memory for stories and letters backward below control levels, again confirming published data (Isaacs et al., 2003). These results might be indicative of weak semantic and sequential immediate recall and working memory (Ramsay and Reynolds, 1995). Verbal memory has been associated with a decreased integrity of the fornix (Nosarti et al., 2014), but further research on the possible changes of the anatomical and functional organization of the fornix, thalamic, and frontal connectivity in preterm children is required.

#### Gray Matter Integrity

Previous studies have shown that cortical thickness is an indicator of the number of neurons per cortical column (la Fougère et al., 2011) as well as of the folding and gyrification of the cortex (Panizzon et al., 2009). In our study, even global cortical thickness was at control levels in the VP-VLBW group, cortical thickness in certain areas was negatively correlated with full scale IQ as well as with some of the subscales whereby the target group was below control levels. This correlation indicates that the thicker the reported areas, the worst performance in full scale IQ and some of its subscales. Previous studies have found disparate results; while some of them showed a positive relation between cortical thickness and IQ (Skranes et al., 2012; Bjuland et al., 2013; Mürner-Lavanchy et al., 2018), others found a negative association (Nam et al., 2015; Sølsnes et al., 2015).

Our results also showed a paradoxically larger right entorhinal area while some other studies show reductions in cortical thickness or surface area of the entorhinal cortex (Skranes et al., 2012, 2013). One potential explanation for this discrepancy is the different age of samples, and the possible atypical cortical development in terms of timing and patterns in preterm entorhinal cortex.

Although the relationship between cortical thickness and cognitive status is not completely defined, some degree of thinning is expected in the cerebral cortex during development. Our data suggest that cortical thinning is occurring in less degree in some areas of the cortex in the VP-VLBW group, which could explain, at least in part, the lower IQ level. This finding requires further research with longitudinal studies to obtain the full picture of the cortical development of the preterm brain.

In line with previous studies with preterm or late-preterm infants (Munakata et al., 2013), children (Reiss et al., 2004; Rogers et al., 2014), adolescents (Nagy et al., 2009), and adults (Pascoe et al., 2019), our study shows also a smaller volume in total GM, subcortical GM (Taylor et al., 2011), thalamus (Nosarti et al., 2008; Martinussen et al., 2009; Bjuland et al., 2014), and fusiform gyrus (Nosarti et al., 2008), as well as enlarged lateral ventricles (Nosarti et al., 2002; Allin et al., 2004; Bjuland et al., 2014) relative to control levels. We also found smaller volume in left ventral diencephalon that includes hypothalamus, basal forebrain, sublenticular extended amygdala and ventral tegmentum. Given the importance of these structures in emotional, social, reward processing, this may explain, at least in part, the frequent need of psychiatric assistance in children born preterm as reported in both large cohort studies (Larroque et al., 2008; Moore et al., 2012).

#### White Matter Integrity

In line with previous studies in VP-VLBW neonates (Miller et al., 2002), infants (Partridge et al., 2004; Lean et al., 2019), children (Nagy et al., 2003; Constable et al., 2008; Young et al., 2019), adolescents (Thomas et al., 2005; Skranes et al., 2007; Mullen et al., 2011), and young adults (Eikenes et al., 2011; Pascoe et al., 2019), our study shows significant decreased FA and increased RD in higher order association areas of the such as frontal, temporal and parietal regions and in the diencephalic WM. As AD and MD appeared affected in the preterm group, this decreased WM integrity might be due, therefore, to increased diffusivity in the mean perpendicular axis (λ2 and λ3). Although the precise mechanisms of WM injury in prematurity are still unclear, global delay in glial maturation after premature brain injury has been previously reported (Salmaso et al., 2014). Animal model data support the hypothesis that gliogenesis, oligodendrocytes and myelin may be at the root of the changes seen in WM integrity in prematurity. In normal development, myelin commences to be visible after 24 weeks of gestation and progress until 36 gestational weeks (Counsell and Boardman, 2005). This supports the hypothesis that migratory pathways from ventricular zone to cerebral cortex and cortico-diencephalic and cortico-striatal connections may be vulnerable to prematurity and other insults occurring during this prenatal period. Further research is needed in the normal and abnormal development of these WM pathways.

#### Limitations of the Study

We acknowledge several possible limitations in the present study. One factor that may be contributing to different results in brain-behavior studies of preterm children is the different inclusion/exclusion criteria. Here we examined children who were born very preterms and had VLBW, but who could be considered relatively ‘healthy’ as they had neither brain injuries detectable by ultrasound at birth nor by MRI at school age. The design of the study excluded children with any form of brain abnormality.

This made our sample very restrictive and may be biased toward the high performance. However, despite of this, the sample of our children’s IQ was still below about 10 points below control level. We believe this is a robust result and consistent with the literature. Nevertheless, our results are optimistic in terms of spared functions in at risk prematurity, and contribute to define the phenotype of this population. This study calls for further research on whether there is a specific cognitive profile in prematurity with some cognitive functions altered and some other spared.

The necessary high quality MRI data (i.e., no movement artifacts) reduced the size of our sample, and this is another limitation: the small number of participants, especially in the control group. This was compensated by using multiple (5000) permutations in FSL, a stringent multiple correction method for false discovery. Brain injuries cause methodological problems in terms of reliability of the registration process of gray and WM analysis, and therefore, we use stringent inclusion criteria (i.e., no neurological impairment or brain damage) to address this and secured the quality of the data.

Further analysis with larger numbers of participants and longitudinal studies will allow more accurate results on the influence of clinical variables on neonatal brain damage and later neuropsychological development.

## Conclusion

In conclusion, the interplay between different perinatal variables has a clear association with brain damage as identified with ultrasound in new born babies with gestational age ≤32 and birth weight ≤1500 g. Selective increased of cortical thickness in higher order processing areas of the frontal, perisylvian, sensory motor and visual cortices is associated with specific components of lower IQ scores in the preterm group. Furthermore, decreased integrity of WM tracts is suggestive of higher order cognitive processing deficits. Identifying these specific neurocognitive deficits might be of help to design interventions aimed to improve academic achievements and mental health, and ultimately, adaptability later in life to society.

## Data Availability

The datasets generated for this study are available on request to the corresponding author.

## Ethics Statement

This study was reviewed and approved by the Ethics Committee of the University of Castilla-La Mancha Medical School and the Albacete University Hospital clinical research committee. Written informed consent was obtained from all the participants and their parents.

## Author Contributions

MC-P contributed to the white matter analysis and writing of the manuscript. RG-M contributed to the data collection and analysis of the neonatal cohort. VS-d-P contributed to the analysis of the gray matter. LL contributed in collecting the data from the medical records of the neonatal cohort. EP-H contributed in the designing of the neuropsychological protocol. FM contributed in the acquisition of the MRI scans. AM provided patients and advice on study design. IO assessed the neurology of the participants. PS contributed in the designing of the study and the recruitment of M.Sc. and Ph.D. students. MU-M contributed in the acquisition of the neuropsychological data. JC contributed in the focused analysis of the fornix. CC contributed in tutoring MC-P during her stay for learning DTI processing. MM-L designed the study, prepared the manuscript and figures for publication, supervised all the students involved, granted the funding, and analyzed the neuropsychological data and the brain MRI alongside the students.

## Conflict of Interest Statement

The authors declare that the research was conducted in the absence of any commercial or financial relationships that could be construed as a potential conflict of interest.

## Footnotes

1 http://surfer.nmr.mgh.harvard.edu/

## Abbreviations

AD: axial diffusivity
BASC: behavior assessment system for children
BW: birth weight
cc: *Corpus callosum*
CI: confidence interval
CPT: Conners’ continuous performance test
DTI: diffusion tensor imaging
FA: fractional anisotropy
Fi: fimbria
Fx: fornix
GA: gestational age
GM: gray matter
ICV: intracranial volume
IQ: intelligence quotient
MD: mean diffusivity
mPFC: medial prefrontal cortex
MRI: magnetic resonance imaging
NEC: necrotizing enterocolitis
NICU: neonatal intensive care unit
PCA: principal component analysis
RD: radial diffusivity
ROP: retinopathy of prematurity
SD: standard deviation
TBSS: tract-based spatial statistics
TOMAL: test of memory and learning
VP-VLBW: very preterm-very low birth weight
WISC: Wechsler intelligence scale for children
WM: white matter.
